# Genetic Structure and Population Affinities of Albanians: A Regional STR-Based Analysis Within the European Context

**DOI:** 10.3390/genes17091059

**Published:** 2026-08-31

**Authors:** Merita Xhetani, Ela Zaimi, Dana Dojčáková, Ilir Sheraj, Soňa Mačeková

**Affiliations:** 1 Department of Biology, Faculty of Natural Sciences, University of Tirana, 1001 Tirana, Albania; 2 Faculty of Technical Medical Sciences, Western Balkans University, 1001 Tirana, Albania; 3 Department of Biology, Faculty of Humanities and Natural Sciences, University of Presov, 08001 Presov, Slovakiasona.macekova@unipo.sk (S.M.); 4 Independent Researcher, 1001 Tirana, Albania

**Keywords:** genetic distances, autosomal STR markers, population structure, Albania

## Abstract

**Background/Objectives**: The population genetic structure of Albania remains insufficiently characterized at both regional and broader European scales. This study investigated genetic variation within Albania and its relationship with neighboring and European populations using autosomal short tandem repeats (STRs). **Methods**: Sixteen autosomal STR loci were analyzed in 2000 unrelated individuals from 12 Albanian counties, grouped into Northern, Central, and Southern regions. Population structure was assessed using principal component analysis (PCA), pairwise Weir–Cockerham FST, hierarchical AMOVA, and Bayesian clustering (STRUCTURE Version 2.3.4). Population affinities were further evaluated using Nei’s standard genetic distance, multidimensional scaling, and neighbor-joining analysis of European reference populations. **Results**: PCA revealed extensive regional overlap, with PC1 and PC2 explaining 2.64% and 2.61% of variation, respectively. Pairwise FST values were close to zero, with confidence intervals overlapping zero, while AMOVA indicated negligible regional differentiation. STRUCTURE identified K = 3 as the strongest relative solution, but ancestry coefficients showed extensive admixture without discrete regional clustering. European comparisons revealed low Nei distances (approximately 0.004–0.014), with Albania showing particularly close affinity to Greece and other Southeastern European populations. **Conclusions**: Albanian autosomal STR variation demonstrates high regional homogeneity and limited population substructure, while broader affinities are consistent with geographic patterns across Southeastern Europe.

## 1. Introduction

The genetic structure of human populations is shaped by a complex interplay of historical, demographic, and evolutionary forces. Patterns of migration, isolation, admixture, and cultural continuity leave identifiable signatures in allele frequency distributions, allowing measurements of genetic distance to serve as powerful tools for reconstructing population history [1,2]. In this context, the study of genetic relationships between populations provides insight not only into their biological affinities but also into the historical events that contributed to their formation.

The Albanian population represents an interesting population genetic study, given its geographic position at the intersection of the Balkan Peninsula and the Mediterranean and its historical interactions with neighboring European populations. Albanians are generally considered descendants of ancient Paleo-Balkan populations, commonly associated with the Illyrians and related groups that inhabited the Western Balkan prior to Roman expansion, although the precise nature of this continuity remains a subject of ongoing historical and linguistic debate [3,4]. Throughout antiquity and the medieval period, the region was influenced by successive Roman, Byzantine, Slavic, Venetian, and Ottoman administrations, creating opportunities for cultural exchange and population interaction. Despite these historical processes, the Albanian language has persisted as a distinct branch of the Indo-European language family, suggesting substantial demographic continuity within the Western Balkan region [1]. The mountainous geography of Albania contributed to the preservation of local communities and regional identities, while limiting extensive population replacement during several historical periods. Following the establishment of the modern Albanian state and particularly during the post-World War II communist era (1945–1990), Albania experienced prolonged political and geographic isolation that substantially reduced migration and external gene flow compared with many neighboring European populations [3,5,6]. Because of this fact, the Albanian population provides a valuable framework for investigating long-term genetic continuity, regional population structure, and genetic affinities within the Balkans.

Comparative analyses of allele frequencies in Albanian populations and neighboring Balkan groups have consistently supported the hypotheses of common ancestry, genetic drift, and historical gene flow. Studies of highly polymorphic HLA loci demonstrate close genetic affinities between Albanians and geographically close populations such as Greeks, Macedonians, Bulgarians, and Italians, reflecting regional continuity and historical interactions [7]; however a different study highlights that the Albanian population exhibits distinct patterns in Rh blood group distribution, characterized by a low prevalence of the weak D phenotype and a specific distribution of the three most common variant alleles within this Caucasian group [8].

Autosomal short tandem repeat (STR) markers have been widely used for population genetic analyses throughout Southeast Europe and have proven particularly useful for investigating genetic relationships among Balkan populations. Numerous studies have characterized STR variation in Kosovo Albanians, Serbians, Croatians, populations of the Dalmatian islands, Montenegrins, North Macedonians, and Turkish populations, demonstrating low levels of genetic differentiation and substantial allele sharing across the region [9,10,11,12,13,14,15,16,17,18,19,20,21]. These investigations have shown that autosomal STR markers are highly informative for estimating genetic distances, reconstructing population affinities, and evaluating patterns of historical connectivity across the Balkans and adjacent regions. However, despite the strategic geographic position of Albania and its importance for understanding Balkan demographic history, comprehensive autosomal STR datasets from large Albanian population samples remain relatively limited. As a result, expanding allele frequency databases and examining the genetic relationships of Albanian regional populations within a broader European and Balkan context can contribute valuable insights into both forensic genetics and population history.

In this study, we evaluated the genetic relationships among three geographically defined regions of Albania—Northern, Central, and Southern—to assess the degree of internal population structure and genetic differentiation within the country. Allele frequency data obtained from 16 autosomal STR loci were analyzed to evaluate forensic and population genetic parameters and to compare patterns of genetic variation across the three regions. Furthermore, population affinities were investigated through genetic distance analyses and comparative assessments with selected European and Balkan populations using statistical software R. The results provide a comprehensive autosomal STR reference dataset for the Albanian population and contribute to a better understanding of its genetic diversity, population structure, and genetic relationships within the broader European context.

## 2. Materials and Methods

### 2.1. Sample and STR Marker Analysis

The samples were collected from individuals originating from all 12 counties of Albania. Information regarding urban or rural residence was not available for all participants and was therefore not included in the analyses. The samples used were the same as described in a previous article by our research group [22]. Certain analyses of autosomal STR markers were conducted for the first time in this study to maintain the coverage of all the regions of Albania. STR typing was done according the same methodology as described at our previous article [22]. Briefly, genomic DNA was extracted from saliva swab samples (Thermo Fisher Scientific, Carlsbad, CA, USA) using the QIAamp DNA Investigator Kit (QIAGEN, Hilden, Germany). Genotyping was performed using the AmpFLSTR™ NGM SElect™ PCR Amplification Kit (Thermo Fisher Scientific, Waltham, MA, USA), a five-dye multiplex assay that simultaneously amplifies 16 autosomal short tandem repeat (STR) loci (D3S1358, vWA, D16S539, D2S1338, D8S1179, D21S11, D18S51, D19S433, TH01, FGA, D10S1248, D22S1045, D2S441, D1S1656, D12S391, and SE33) [23] together with the sex-determining marker Amelogenin. This kit was selected because it represents one of the most widely adopted forensic STR systems in Europe and was specifically developed to meet the recommendations of the European Network of Forensic Science Institutes (ENFSI) and the European DNA Profiling Group (EDNAP). The marker panel includes the expanded European Standard Set of Loci (ESSL), ensuring compatibility with European forensic databases and facilitating comparative population genetic analyses across European and Balkan populations.

### 2.2. Data Distribution and Analysis

For population-level analyses, the 12 Albanian counties were grouped into three broad geographic regions: Northern, Central, and Southern Albania. This classification does not correspond to an official administrative division but reflects a commonly used geographic and demographic framework in studies of Albanian population structure, settlement patterns, and internal migration. The three-region grouping is supported by major physiographic features, including mountain systems, river basins, and historical communication routes, which have influenced patterns of population mobility and interaction throughout the country’s history. Northern Albania is characterized by a predominantly mountainous landscape and historically more isolated communities, whereas Central Albania encompasses the principal urban and economic corridor of the country, including the Tirana–Durrës region. Southern Albania has historically maintained stronger connections with the Southern Balkans and the Mediterranean through trade, migration, and cultural exchange. Contemporary demographic studies have also documented distinct migration dynamics among these regions, particularly the large-scale population movement from northern and southern rural areas toward central urban centers following the political and economic transition of the 1990s [24]. The use of these three macro-regions therefore provides a geographically and demographically meaningful framework for evaluating potential patterns of genetic differentiation while maintaining adequate sample sizes for statistical analyses.

**Figure 1:**
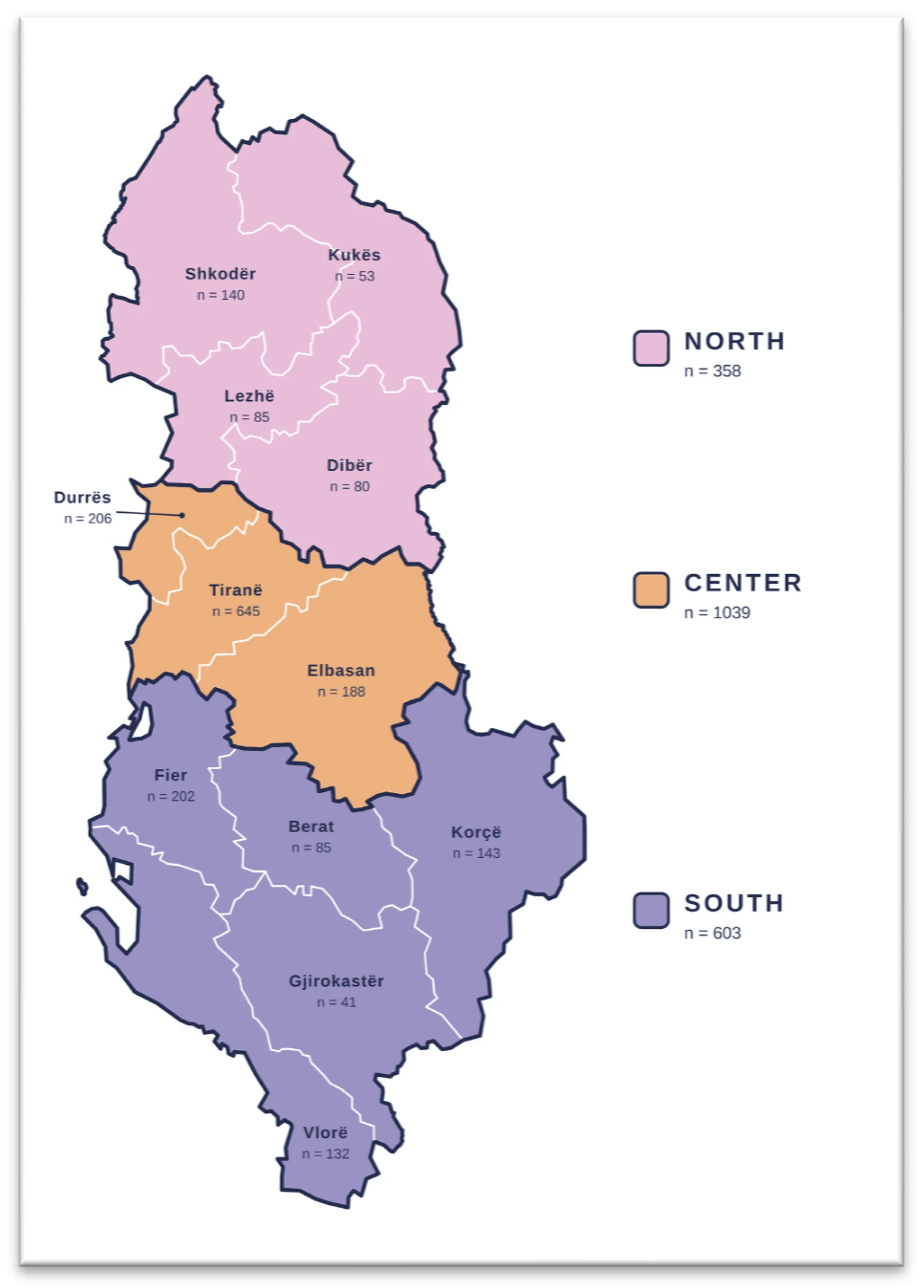
The three geographic regions of Albania with the top administrative divisions, their main cities, and the number of samples analysed for each based on the number of inhabitants per counties—data published from INSTAT year 2021 [25].

The 12 counties with the main cities representing them and the number of samples per each are presented in Figure 1.

Tirana is the capital and the largest city of Albania, the country’s most densely populated urban center, and in this study it is represented with of 645 samples, followed by Durrës with 206 samples, Fier with 202 samples; Elbasan with 188 samples; Korçë with 143 samples; Shkodër with 140 samples; Vlorë with 132 samples; Berat and Lezhë with 85 samples respectively; Dibër with 80 samples; Kukës with 53 samples; and Gjirokastër with 41 samples.

#### 2.2.1. Region-Wise Exploration

To investigate within-country population structure, individuals were assigned to three broad geographic regions according to their city of origin: North (Shkodër, Kukës, Dibër, and Lezhë), Center (Tiranë, Durrës, and Elbasan), and South (Fier, Korçë, Berat, Vlorë, and Gjirokastër) (Figure 1). These regions largely reflect the major Gegë- and Tosk-speaking areas of Albania, with the northern region corresponding predominantly to the Gegë-speaking area and the central and southern regions falling largely within the Tosk-speaking area. This geographic distinction is relevant to the interpretation of population structure because the Gegë–Tosk division represents an important historical and cultural differentiation within Albania. The resulting regional groups comprised 358, 1039, and 603 individuals, respectively.

All analyses were performed on diploid multilocus genotypes from the 16 autosomal STR loci using R version 4.6.1 and the adegenet, hierfstat, poppr version 2.9.7 [26] and ade4 packages version 1.7-23 [27].

Principal component analysis (PCA) was used as a model-free exploratory assessment of regional genetic structure. The proportion of total genetic variance explained by each principal component was calculated from the corresponding eigenvalues, and individual scores on the first two components were visualized according to geographic region.

Regional differentiation was quantified using pairwise Weir–Cockerham FST estimates [28]. The genind genotype object was converted to the hierfstat format using genind2hierfstat, and pairwise estimates were calculated with pairwise.WCfst. Sampling uncertainty was evaluated by nonparametric locus bootstrap. In each of 500 bootstrap replicates, the 16 STR loci were sampled with replacement, pairwise FST values were recalculated, and the resulting bootstrap distributions were summarized by their medians and percentile-based 95% confidence intervals. A hierarchical analysis of molecular variance (AMOVA) was conducted to partition genetic variation among regions, among cities nested within regions, among individuals within cities, and within individuals [29].

#### 2.2.2. STRUCTURE Analysis

Individual-level population structure was also investigated using the Bayesian clustering algorithm implemented in STRUCTURE version 2.3.4 [30]. The number of assumed genetic clusters was evaluated from K = 1–6, with 20 replicate runs per K. Each run used a burn-in period of 100,000 Markov chain Monte Carlo iterations (BURNIN = 100,000), followed by 500,000 sampling iterations (NUMREPS = 500,000), with six as the maximum number of populations evaluated (MAXPOPS = 6). Individual ancestry membership coefficients (Q) were extracted and visualized using the R package pophelper version 2.3.1.

The estimated log probability of the data, lnP(D∣K), was summarized across the 20 replicate runs at each value of K. The Evanno ΔK statistic, which measures the standardized second-order rate of change in likelihood between successive values of K, was calculated using evannoMethodStructure [31]. Both the mean likelihood and ΔK were considered when evaluating the clustering results, together with the consistency of replicate runs and visual inspection of individual Q-membership coefficients. Because ΔK cannot be calculated for K = 1, its maximum was interpreted as the strongest relative subdivision among the forced K ≥ 2 solutions rather than as an independent test against the absence of population subdivision.

#### 2.2.3. Population-Wise Genetic Distance Analysis

Population allele frequency data were obtained from the STRidER database [32] and imported into R. Database-wide and continental aggregate entries, together with populations outside the geographic scope of the study, were excluded prior to analysis. The retained STRidER dataset comprised 22 European reference populations: Austria, Belgium, Bosnia and Herzegovina, Croatia, the Czech Republic, Denmark, Finland, France, Germany, Greece, Hungary, Ireland, Italy, Montenegro, the Netherlands, Norway, Poland, Slovakia, Slovenia, Spain, Sweden, and Switzerland. Published allele frequency datasets representing Albania, Kosovo, Turkish Cyprus, and the Aegean and Mediterranean regions of Turkey were subsequently incorporated. Serbia as Balkan population does not have an available dataset and was left outside the comparison. Two datasets were constructed according to marker availability. The first included all populations and was restricted to the 10 STR loci shared across datasets, whereas the second excluded Kosovo and Turkish Cyprus, allowing analysis of 15 shared loci. Prior to the analysis, allele labels and frequencies were standardized, duplicate allele entries were combined by summing their frequencies, and allele frequencies at each locus were normalized to sum to one.

Pairwise genetic differentiation among populations was quantified using Nei’s standard genetic distance. For each pair of populations, allele frequency vectors were aligned across the union of alleles observed at each locus, with alleles absent from one population assigned a frequency of zero. Nei’s genetic identity was calculated across the shared loci as (I = J_{XY}/\sqrt{J_XJ_Y}), where (J_{XY}) represents the summed products of corresponding allele frequencies and (J_X) and (J_Y) represent the summed squared allele frequencies of the two populations, respectively. Nei’s standard genetic distance was then calculated as (D = −\ln(I)), and the resulting pairwise values were assembled into a symmetric genetic distance matrix. Population relationships were visualized using neighbor-joining (NJ) trees constructed from the Nei distance matrices with the function of the R package ape version 5.8-1 [33]. Branch support was assessed by nonparametric bootstrap resampling of loci. In each of 500 bootstrap replicates, the complete set of shared loci was sampled with replacement, a new Nei distance matrix was calculated, and an NJ tree was reconstructed. Bootstrap clade support was calculated as the percentage of replicate trees containing each clade of the reference NJ tree using prop.clades in ape. The complete procedure was applied independently to the 10-locus and 15-locus datasets.

All analyses were performed in R computing environment, Version 4.6.1, The code for replicating the entire workflow is available online (see Data Availability Statement).

## 3. Results

### 3.1. Distribution of Data for Three Main Regions

Allele frequencies were calculated using data obtained from the DNA analysis of 2000 unrelated individuals. Amelogenin analysis identified 1370 males (68.5%) and 630 females (31.5%) among the 2000 individuals analyzed. All samples yielded interpretable amelogenin profiles. Allele frequency distributions for all 16 autosomal STR loci are presented in Supplementary Table S1. A total of 4000 alleles from 2000 unrelated Albanian individuals were analyzed, providing the population reference dataset used for subsequent forensic and population genetic analyses.

**Figure 2:**
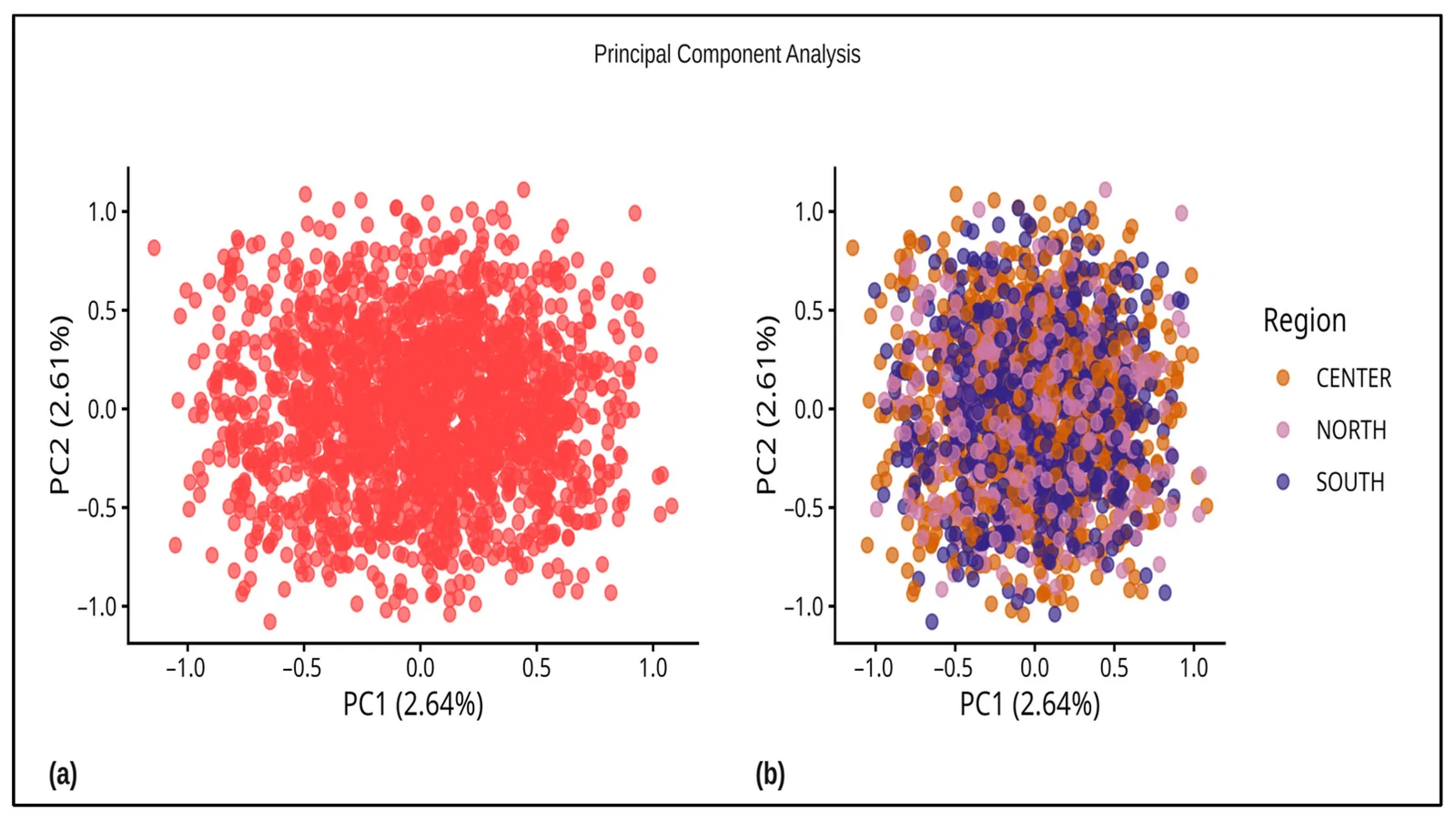
(**a**) Principal component analysis (PCA) plot of the Albanian population based on autosomal STR markers. Each dot represents an individual genotype. (**b**) Principal component analysis (PCA) of the Albanian population, stratified by geographic region (Center, North and South). Each dot represents an individual, colored according to regional origin.

Principal component analysis (PCA) based on autosomal STR markers revealed a largely homogeneous genetic structure within the Albanian population (Figure 2a,b). The analysis showed extensive overlap among individuals from different regions, with no evidence of distinct genetic clustering. Most individuals cluster tightly around the center of distribution, indicating limited genetic differentiation and a high degree of common ancestry. The first principal component (PC1), accounting for 2.64% of the total variance, and the second principal component (PC2), explaining 2.61% of the variance, do not reveal any discernible subpopulation stratification. The compact clustering pattern supports the conclusion that the sampled population is genetically cohesive when assessed using autosomal STR markers, with minimal internal structure. The organization in three non-administrative regions as North Albania includes Shkodër, Kukës, Dibër and Lezhë with 358 samples in total; Central Albania includes Tirana, Durrës and Elbasan with 1039 in total and South Albania including Vlora, Berat, Fier, Gjirokastër and Korçë with 603 samples in total is showed in Figure 2b. Individuals from all three regions overlap largely within a single, dense cluster, indicating the absence of a clear genetic substructure and suggesting extensive gene flow throughout the country. The first principal component (PC1), explaining 2.64% of the total variance, and the second principal component (PC2), accounting for 2.61% of the variance, do not distinguish between regional groups, further supporting the lack of population stratification. In both PCA plots only a small number of outliers are observed; these are scattered across regions and do not form distinct clusters, possibly reflecting individual-level variation rather than systematic regional differentiation. These results indicates that there is no strong genetic separation between the regions and the differences are very small and not significant.

#### Evidence of Minimal Regional Genetic Differentiation in the Albanian Population

**Figure 3:**
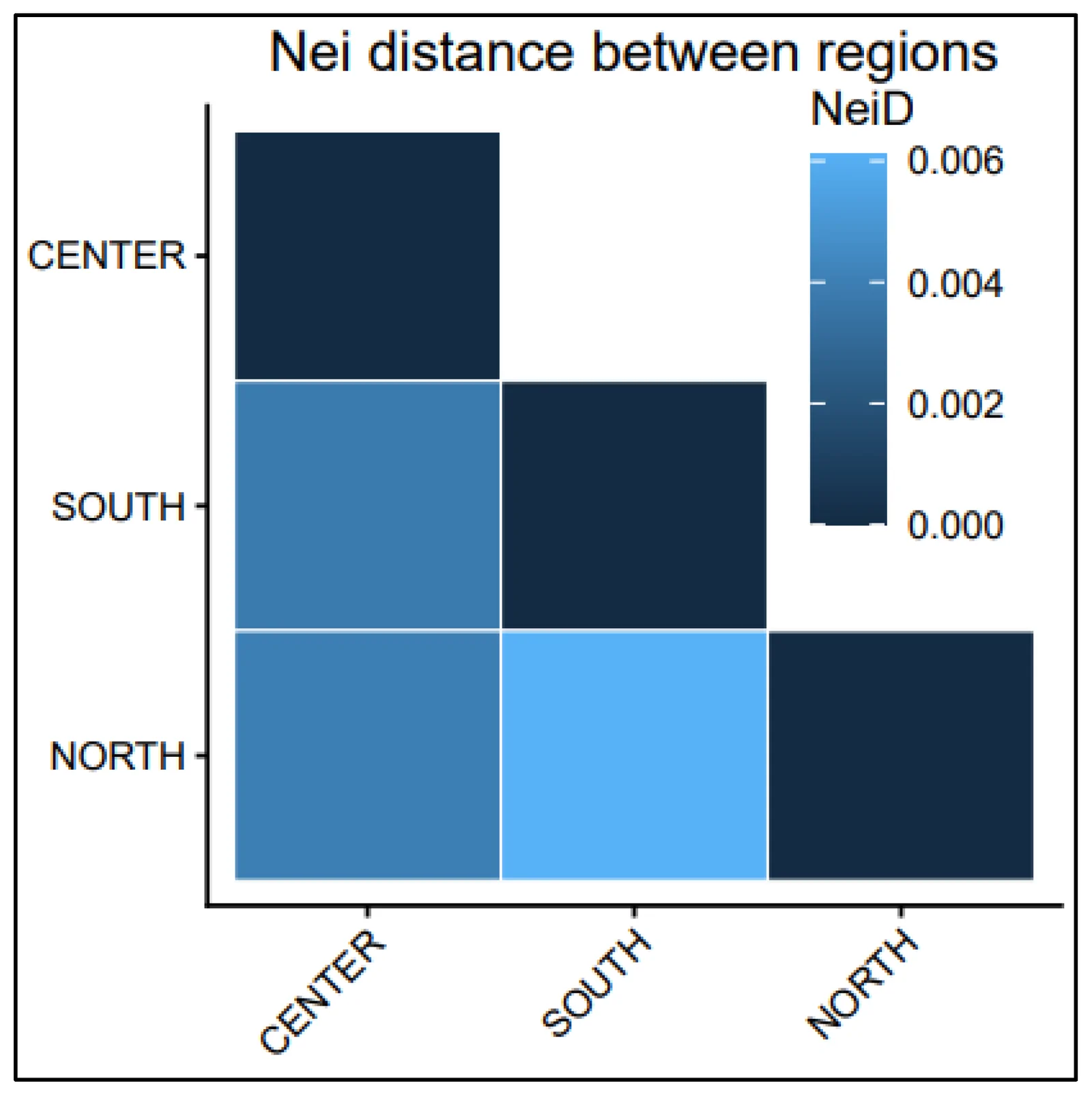
Heatmap of pairwise Nei’s genetic distances among Albanian geographic regions (Center, South, and North) based on autosomal STR markers. Darker shades indicate lower genetic distances and greater similarity, whereas lighter shades represent higher distances and increased differentiation.

**Figure 4:**
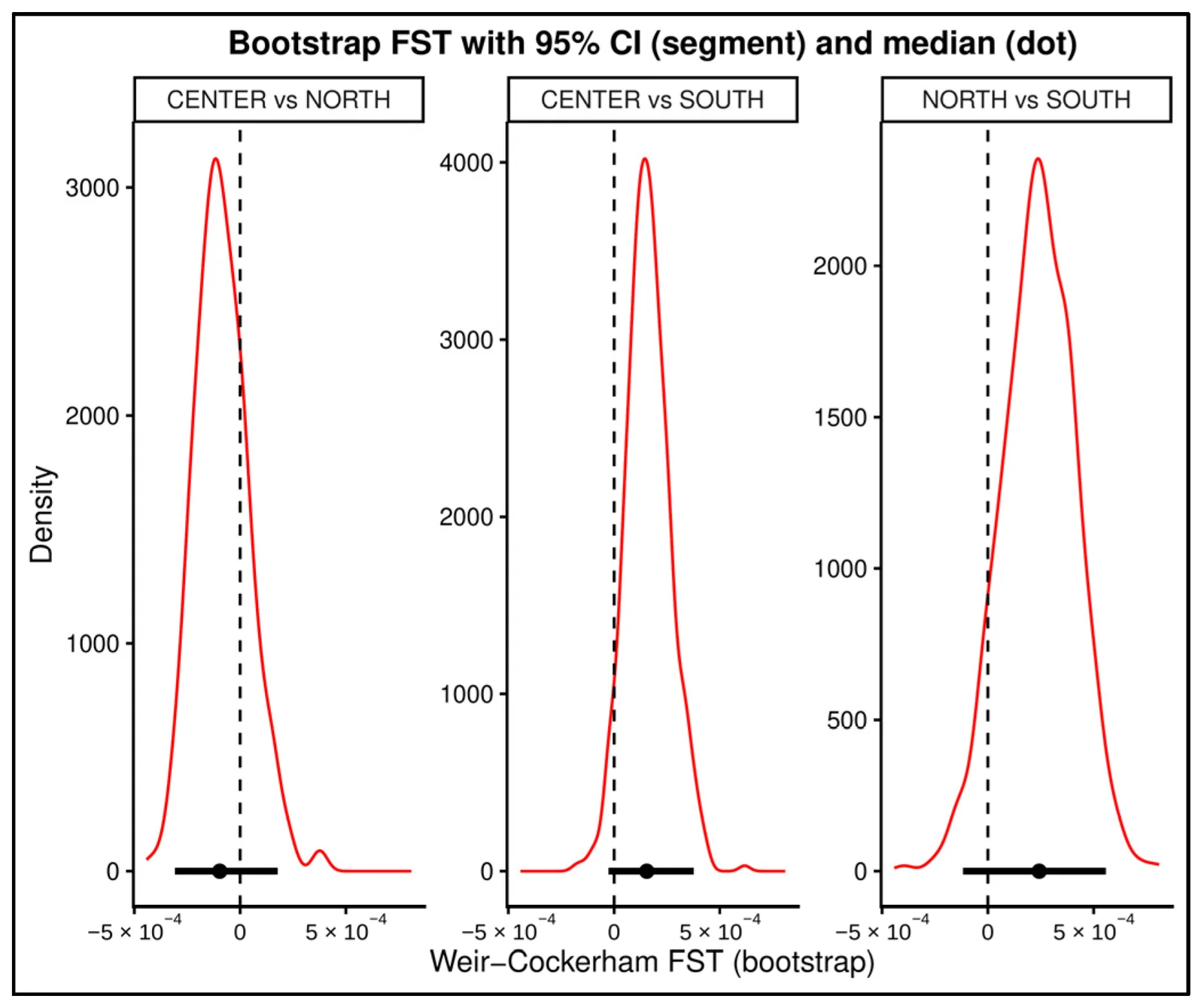
Bootstrap distributions of pairwise Weir–Cockerham F_ST estimates among Albanian geographic regions based on autosomal STR markers. Comparisons shown are Center vs. North, Center vs. South, and North vs. South. Red curves represent the bootstrap density distributions of F_ST values. Black horizontal segments indicate the 95% confidence intervals, and black dots denote the median F_ST estimate for each comparison.

Nei’s genetic distance calculated by comparing the allele frequencies of three regions is shown in Figure 3 and bootstrap distributions of Weir–Cockerham F_ST in Figure 4.

The matrix demonstrates very low overall differentiation among regions, with the closest relationship observed between the Center and South regions. The North region shows slightly higher distances relative to the other two groups, although all values remain low. Bootstrap distributions of pairwise Weir–Cockerham F_ST estimates among the Albanian regional groups (Center vs. North, Center vs. South, and North vs. South) indicate extremely low levels of population differentiation. In all comparisons, the median F_ST values are positioned close to zero, and the 95% confidence intervals broadly overlap zero, suggesting negligible genetic structure among regions. The density curves are narrow and centered around low positive values, consistent with only minor allele frequency differences across regional groups. Among the three comparisons, the North vs. South contrast shows a slightly right-shifted distribution relative to the other pairs, indicating marginally higher differentiation; however, the effect remains very small and biologically limited. These findings support substantial genetic continuity and extensive shared ancestry across the North, Center, and South of Albania, encompassing populations from both the predominantly Gegë-speaking northern region and the largely Tosk-speaking central and southern regions. Despite this historical and cultural Gegë–Tosk differentiation, no evidence of meaningful regional genetic subdivision was observed based on autosomal STR markers.

**Figure 5:**
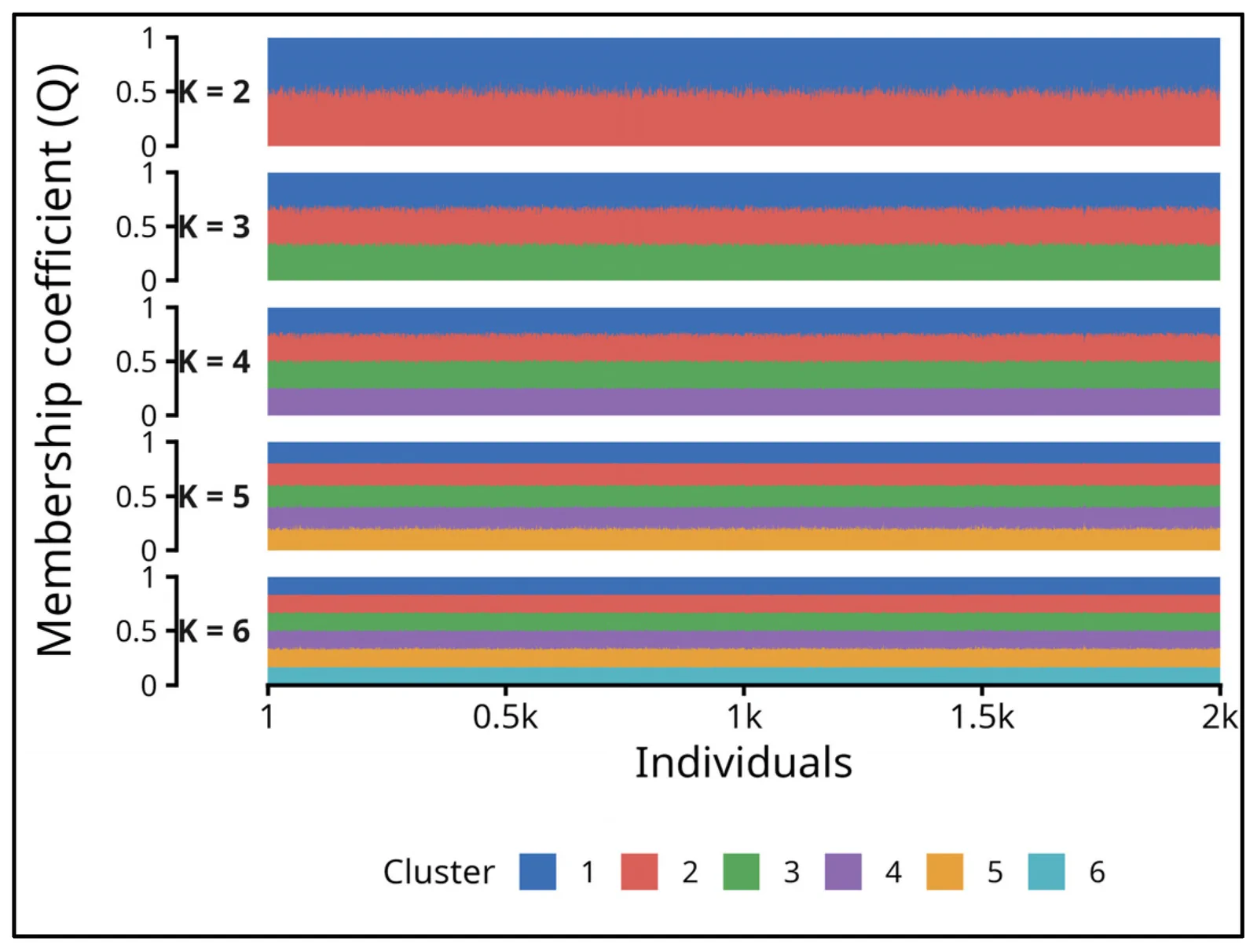
The population structure analysis of studied regions obtained by using the STRUCTURE version 2.3.4 program and results assuming a different number of genetic clusters (K = 6) discrimination. Each individual is represented along the *x*-axis, while the colored proportions indicate that individual’s estimated membership coefficient (Q) in each inferred cluster. The length of the segments is proportional to the estimated proportion in the statistically determined genetic group.

The STRUCTURE analysis further supports the absence of pronounced genetic substructure within the Albanian population (Figure 5). Across all tested values of K (2–6), the ancestry profiles of the 2000 individuals displayed broadly similar proportional contributions from the inferred genetic components, without the emergence of clearly separated or geographically discrete clusters.

Each horizontal panel shows the STRUCTURE result assuming a different number of genetic clusters (K). What to expect if there is heterogeneity: A person assigned strongly to one genetic cluster would therefore be dominated by one color, whereas mixed colors indicate fractional membership in several clusters. There is essentially no discrete clustering of individuals at any tested K. At K = 2, almost every individual is divided approximately equally between the two inferred clusters. The same pattern continues as K increases: at K = 3 individuals receive roughly one-third membership in each cluster, at K = 4 roughly one-quarter, and so forth. There are no obvious blocks of individuals dominated by particular colors and no subset of individuals consistently assigned predominantly to a separate cluster at of all 20 runs.

**Figure 6:**
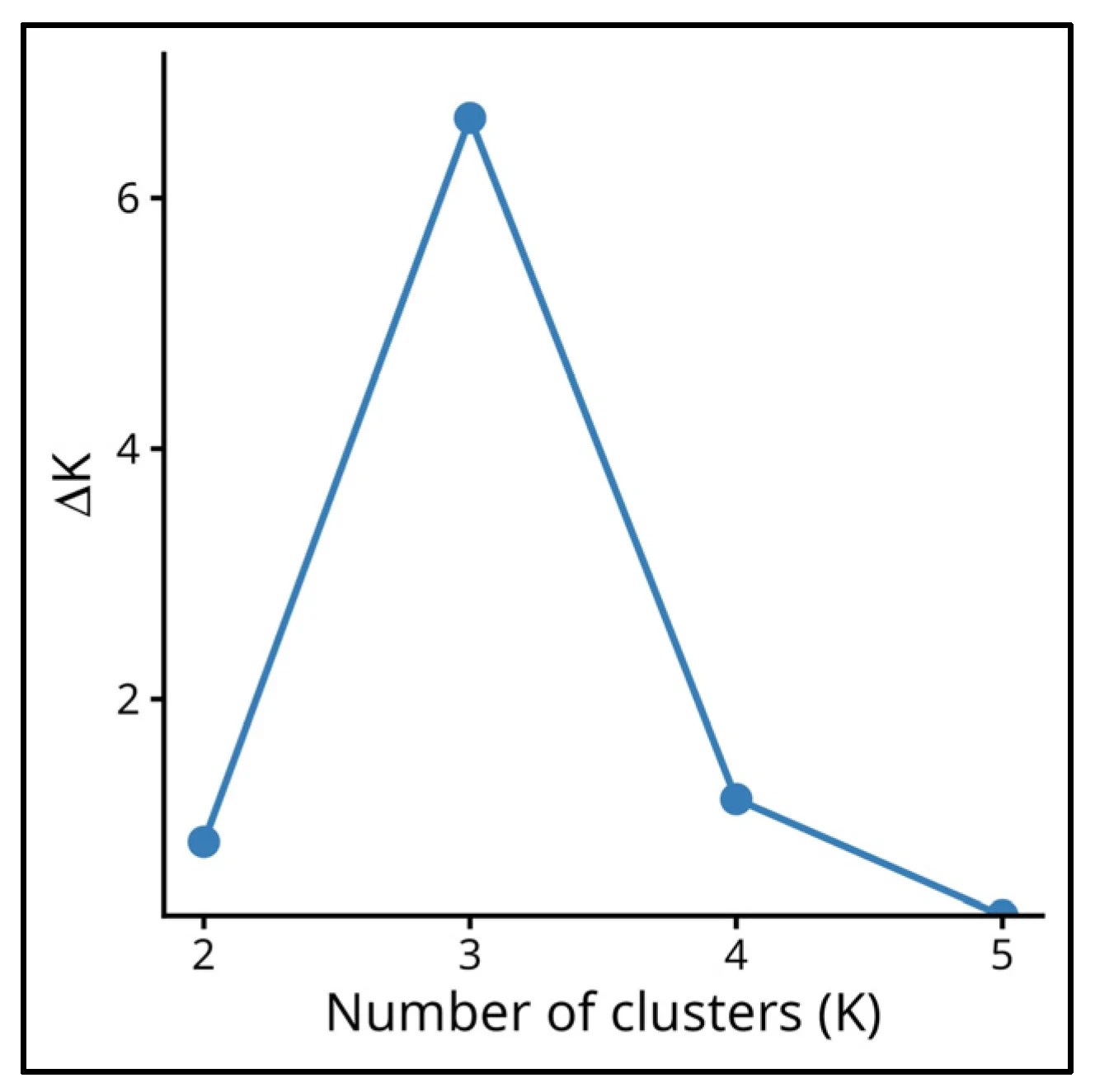
Evanno plot from the STRUCTURE analysis, used to identify the most likely number of genetic clusters in the Albanian population.

The ΔK analysis identified K = 3 as the optimal number of genetic clusters, showing a pronounced maximum at K = 3 compared with K = 2, 4, and 5 (Figure 6).

**Figure 7:**
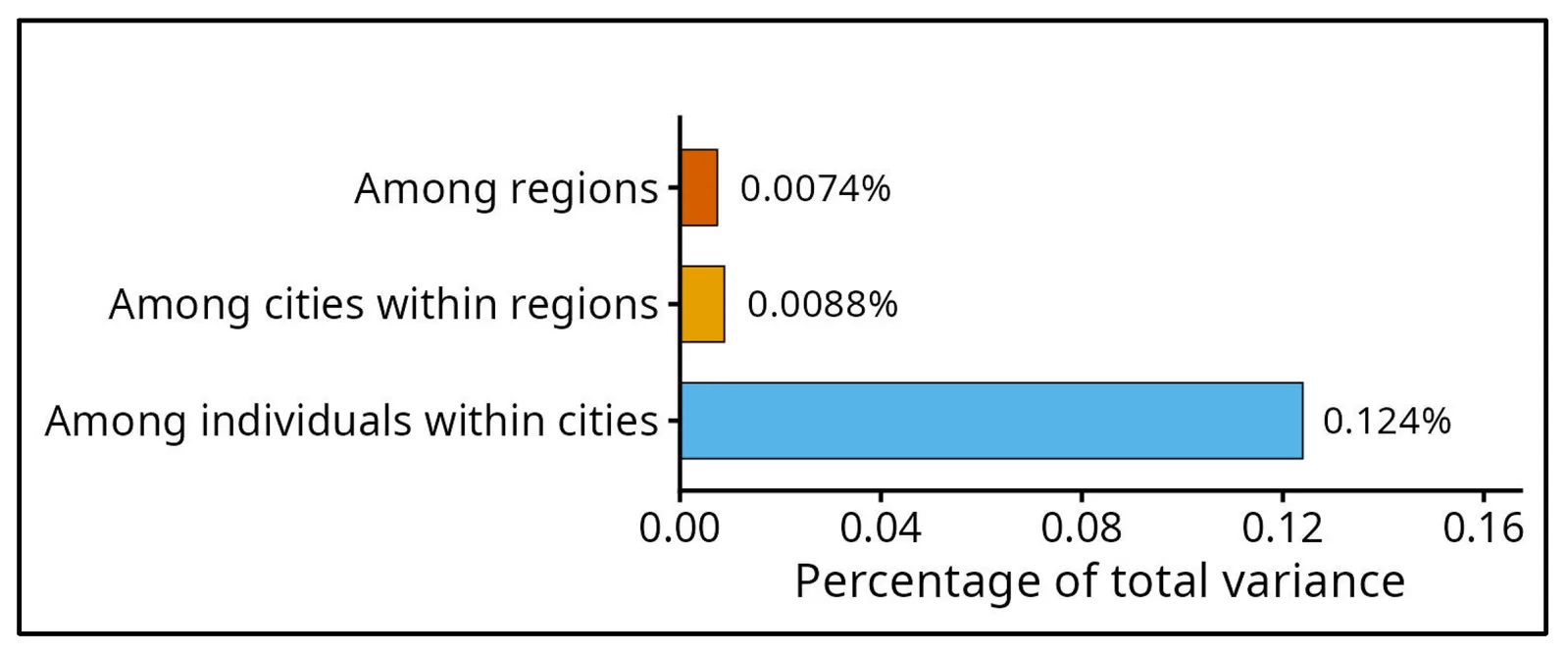
AMOVA partitioning of genetic variance including cities, regions and individuals.

AMOVA suggest only a week degree of population substructure, with substantial admixture and shared ancestry among individuals from Northern, Central and Southern Albania (Figure 7).

Because of the fact that our samples were grouped into Northern Albania, Central Albania and Southern Albania, the peak at K = 3 suggests that the dataset statistically supports the presence of three underlying genetic components. However, in the PCA, pairwise FST analysis, and AMOVA the structure results suggest only a week degree of population substructure, with substantial admixture and shared ancestry among individuals from Northern, Central and Southern Albania.

Summary population genetic statistics for all analyzed STR loci are presented in Supplementary Table S2. These include observed heterozygosity (Ho), average within-population gene diversity (Hs), total gene diversity (Ht), genetic differentiation measures (Dst and Dstp), fixation indices (FST and FSTp), the inbreeding coefficient (FIS), and Jost’s differentiation index (Dest). The consistently high heterozygosity values observed across loci indicate substantial genetic diversity within the Albanian population. In contrast, the very low values of FST, Dst, and Dest demonstrate minimal genetic differentiation among the Northern, Central, and Southern regional groups, supporting the conclusion that the Albanian population is genetically homogeneous and lacks significant internal population substructure.

The complete LD matrices are provided in Supplementary Tables S3–S5. After correction for multiple testing, no consistent patterns of significant linkage disequilibrium were observed, supporting the assumption of locus independence required for forensic calculations.

The exact tests for Hardy–Weinberg equilibrium (HWE), based on 10,000 permutations, are presented in Supplementary Table S7. All loci were generally consistent with HWE expectations after correction for multiple comparisons, indicating the absence of substantial population stratification, inbreeding, or genotyping bias.

### 3.2. Patterns of Genetic Affinity Across European Populations

Next, we calculated genetic distances between studied populations (Table 1).

**Figure 8:**
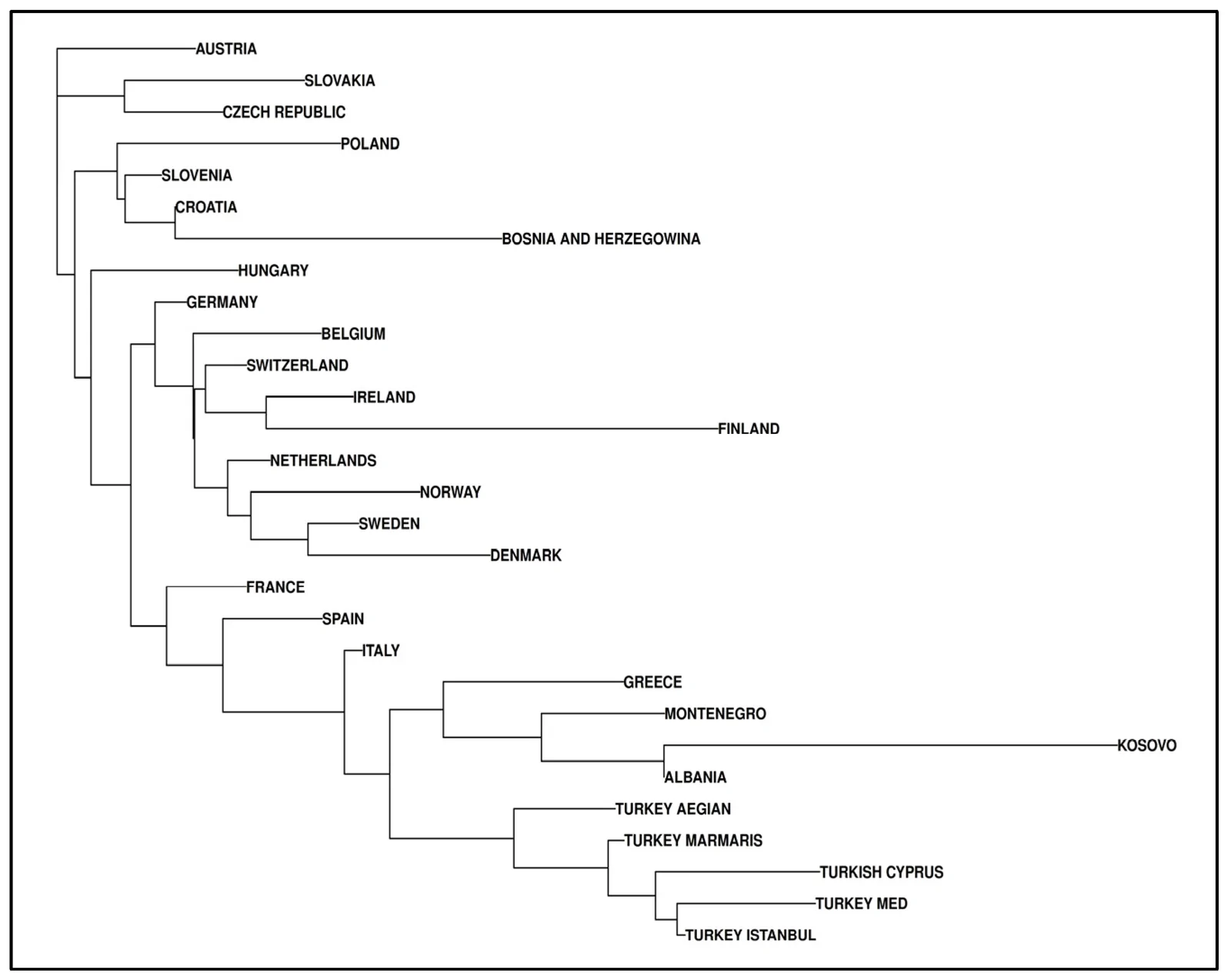
Neighbor-joining tree showing the relationship of Albania population and 28 others populations. The tree was constructed based on allele frequencies for 10 autosomal STR loci shared among all populations (D2S1338, D3S1358, D8S1179, D16S539, D18S51, D19S433, D21S11, FGA, TH01, VWA) [23].

**Figure 9:**
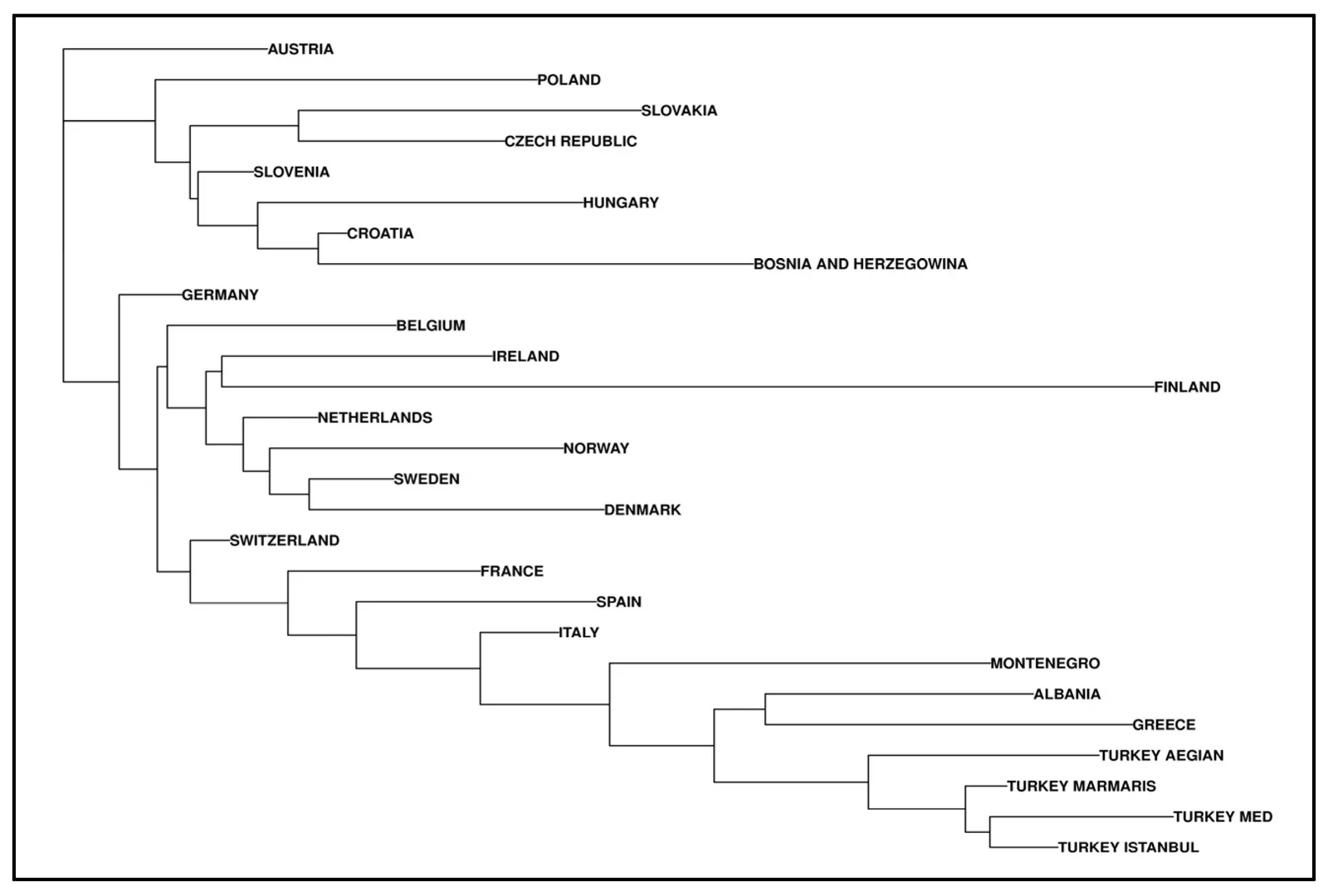
Neighbor-joining tree showing the relationship of Albania population and 26 others populations. The tree was constructed based on allele frequencies for 15 autosomal STR loci shared among all populations (D1S1656, D2S1338, D2S441, D3S1358, D8S1179, D10S1248, D12S391, D16S539, D18S51, D19S433, D21S11, D22S1045, FGA, TH01, VWA) [23].

**Figure 10:**
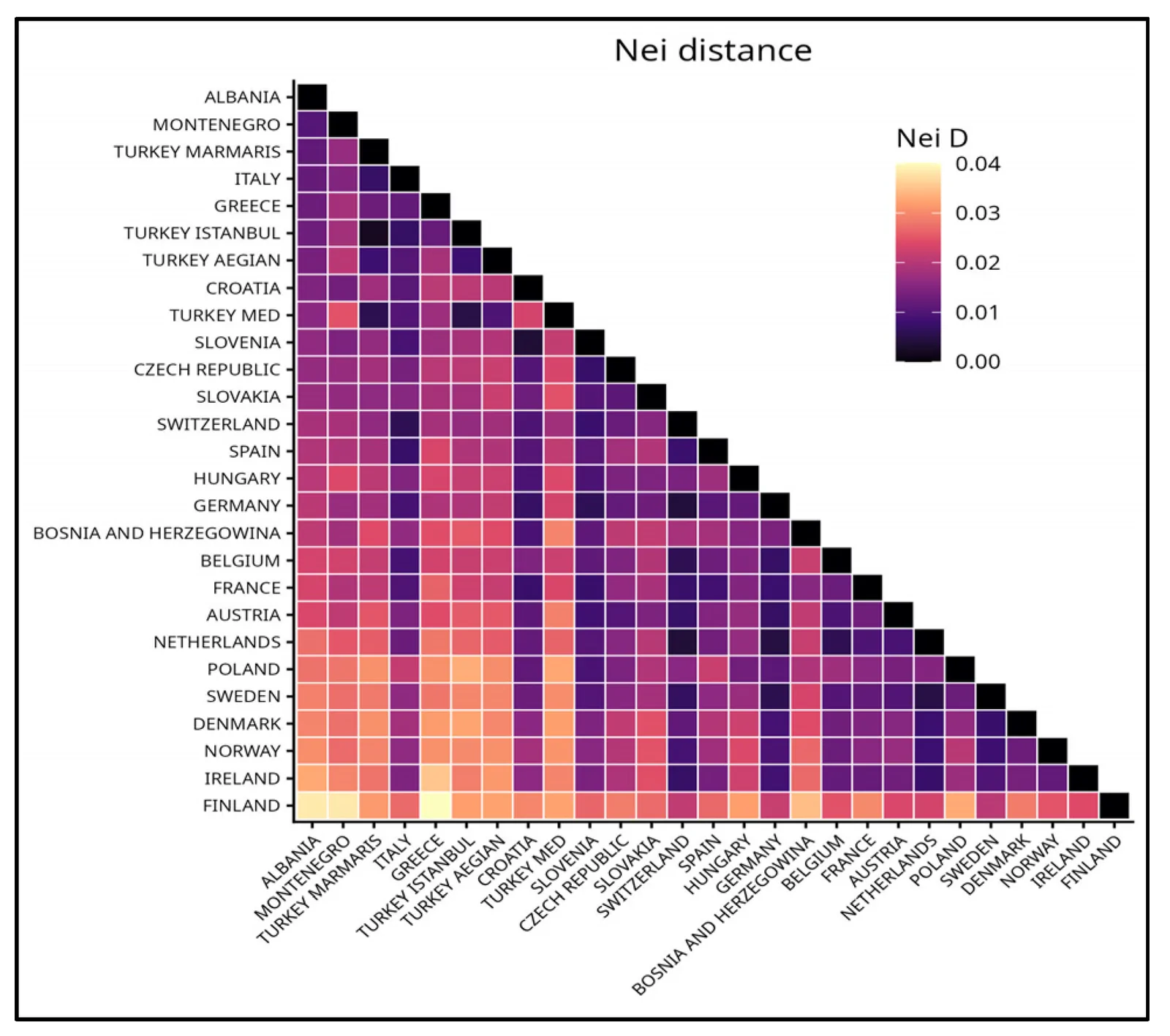
Heatmap of pairwise Nei’s genetic distances among 27 European populations based on 15 autosomal STR markers. Darker shades indicate lower genetic distances and greater similarity, whereas lighter shades represent higher distances and increased differentiation.

A neighbor-joining (NJ) tree based on allele frequency data from 10 autosomal STR loci shared across all 28 European populations is presented in Figure 8. To provide a more comprehensive comparison using a larger set of genetic markers, a second NJ analysis was performed using 15 autosomal STR loci available for 26 European populations. The NJ tree and the corresponding heatmap, based on Nei’s genetic distances between pairs of populations, are shown in Figure 9 and Figure 10, respectively. Thus, Figure 8, Figure 9 and Figure 10 represent complementary analyses: the first maximizes the number of populations included, while the other two maximize the number of shared STR loci and provide a more detailed assessment of genetic distances between populations.

Nei’s genetic distance measures accumulated allele frequency differences between populations. A neighbor-joining tree based on Nei’s genetic distances reveals that the Albanian population is genetically closest to those of Montenegro and Kosovo, forming a distinct Western Balkan cluster. Greece appears as the next closest population, while Italy joins this cluster at a slightly greater genetic distance. This topology indicates a high degree of genetic similarity among Western and Southern Balkan populations, reflecting their shared evolutionary history and geographic proximity.

Albania and Montenegro exhibit the shortest branch lengths among Balkan populations indicating minimal genetic differentiation, while Kosovo joins the Albanian–Montenegrin branch, supporting a close genetic relationship within the Western Balkans. Greece connects to this cluster at a slightly deeper node, suggesting significant affinity. Italy occupies an intermediate position between the Balkan and Western European groups, consistent with Mediterranean genetic connections. Turkish populations form a neighboring yet distinct Eastern Mediterranean cluster, separate from the Western Balkan branch. A notable feature is the length of the branch leading to Kosovo, which appears exceptionally large relative to its position within the cluster; however, verification against the Nei’s distance matrix suggests this is primarily a graphical anomaly.

Pairwise Nei’s genetic distances between the Albanian population and the analyzed European populations are provided in Supplementary Table S6. The Kosovo and Turkish Cyprus samples were left out as they were the only population with 10 available STR loci.

The Albanian population shows its strongest genetic affinities with populations geographically adjacent to the Western Balkans—specifically Greece and Montenegro, followed by Italy.

More distant relationships are observed with populations from the Western, Central, and Northern Europe and Turkish populations form a neighboring yet distinct Eastern Mediterranean cluster, separate from the Western Balkan branch. Within our cluster, studies involving Turkish samples form a distinct Eastern Mediterranean subgroup; Aegean Turkey is closer to the Balkan grouping, while Marmaris, Mediterranean Turkey, Istanbul, and Turkish Cyprus form a distinct branch.

Pairwise Nei’s genetic distances calculated from 15 autosomal STR loci demonstrated low overall differentiation among the 27 populations studied (Figure 10). Albania showed the smallest genetic distances with neighboring Balkan and Eastern Mediterranean populations, whereas larger distances were observed with Northern European populations. Finland displayed the greatest differentiation from most populations included in the analysis. The pattern of pairwise distances suggests substantial genetic continuity across Southern and Central Europe, with increasing divergence toward northern regions of the continent.

## 4. Discussion

The present study provides evidence that the Albanian population exhibits a high degree of genetic homogeneity, with negligible differentiation among the Northern (Gegë), Central, and Southern (Toskë) regions. The absence of detectable genetic subdivision suggests that historical cultural and linguistic boundaries between Gegë and Toskë populations have not resulted in substantial reproductive isolation.

The observed homogeneity is consistent with the demographic history of Albania and the Western Balkans. Although the region has experienced successive prehistoric, Illyrian, Roman, Byzantine, Slavic, and Ottoman influences, these events appear to have promoted admixture rather than genetic fragmentation. Previous Y-chromosomal, mtDNA, and genome-wide studies have likewise demonstrated substantial continuity among Balkan populations and a shared ancestry shaped by repeated migrations and long-term regional interactions [58,59]. Contemporary Albanians represent a relatively cohesive population within the broader Balkan genetic landscape.

Comparative analyses based on Nei’s genetic distances further support this interpretation. The smallest genetic distances were observed between Albanians and Greeks, followed by other Balkan populations, whereas somewhat greater distances were found with Central and Western European populations. The minimal genetic distances observed between Albanian and Greek populations in this study align with previous genetic research reporting close affinities between these groups. Using Nei’s genetic distance based on HLA-A, HLA-B, and HLA-DRB1 allele frequencies, Sulçebe and Shyti [7] reported a close genetic relationship between Albanians and Greeks, with Northern Greeks representing one of the European populations most closely related to Albanians. There is also an earlier study specifically analyzing 15 AmpFlSTR Identifiler loci in Kosovo Albanians by Kubat et al. [16] that reports particularly close genetic affinities with Greeks and other Southern European populations similarities. This study also demonstrated allele frequency patterns characteristic of European populations and provided evidence of their close genetic affinity within the broader structure of the Balkan population.

Autosomal analyses of Western Balkan populations have shown that Albania exhibits particularly close genetic affinities with Greeks and other Southern European populations, thereby supporting the hypothesis of a shared Southeastern European genetic heritage. Similar affinities have been reported in STR-based studies of Greece, Montenegro, Serbia, Croatia, and Bosnia and Herzegovina, where pairwise F_ST and genetic distance values generally remain low and reflect geographic proximity rather than sharp ethnic boundaries [9,11,13,17,19,21,56,60,61]. The intermediate position of Italy is also consistent with previous Mediterranean population studies [62]. The Italian population in this analysis aligns with previous whole-genome studies, Fioreto et al., describes Italy as a genetic crossroads between continental Europe and the Mediterranean, reflecting a complex history of gene flow and population movements [63]. In particular, genetic affinities have been reported between Italian and Balkan populations—including Albanians—supporting the role of the Adriatic region and the broader Mediterranean area as a significant corridor for historical population interaction. Turkish samples form a distinct Eastern Mediterranean subgroup; Aegean Turkey is closer to the Balkan grouping, while Marmaris, Mediterranean Turkey, Istanbul, and Turkish Cyprus form a distinct branch. This pattern is to be expected, as Western Anatolia shares stronger genetic ties with Southeastern Europe than other Turkish regions [64].

The gradual increase in Nei’s genetic distance from the Balkans toward Northern Europe supports the existence of geographic structuring of genetic diversity across Europe. The relatively low distances observed between Albania and neighboring Balkan populations are consistent with long-term regional connectivity and shared demographic histories.

Greater genetic distances were observed between the Albanian population and certain Northern European populations. Finland exhibited the greatest genetic differentiation from the majority of populations included in the analysis. This pattern aligns with previous population genetic studies showing that Finnish populations occupy a relatively distinct position within the European genetic landscape. Genome-wide analyses have demonstrated increased differentiation and reduced genetic diversity in Finnish populations compared to Central European populations—a phenomenon attributed to founder effects, population bottlenecks, relatively small historical population size, and prolonged geographic isolation. Similar patterns of regional differentiation within Finland, that study of Salmela et. al., and Palo et al., have also been documented using Y-chromosome STRs and other genetic markers, reflecting the distinctive demographic history of the Finnish population [65,66].

The consistent patterns observed in the supplementary NJ analyses despite differences in the number of populations and shared STR loci support the robustness of the inferred relationships between populations and indicate that the observed genetic affinities are not significantly influenced by variation in marker availability.

The population genetic parameters obtained in the present study are comparable to those reported for neighboring Balkan and Near Eastern populations. High observed heterozygosity values and low fixation indices are consistent with findings from Greek, Turkish, Croatian, and Montenegro STR databases, all of which demonstrate substantial genetic diversity and high discriminatory capacity for forensic applications [67]. The absence of significant deviations from Hardy–Weinberg equilibrium and the lack of consistent linkage disequilibrium after correction for multiple testing further support the suitability of these loci for forensic calculations. From a forensic perspective, the extremely low levels of regional differentiation have important practical implications. Since allele frequencies are effectively uniform across Albania, a single national allele frequency database can be reliably applied for forensic casework, paternity testing, and kinship analyses without introducing substantial bias due to population substructure. This finding aligns with recommendations from population studies conducted in neighboring Balkan populations, where similarly low F_ST values have justified the use of national reference databases [68]. Overall, the combination of high within-population diversity, negligible regional differentiation, and close affinities with neighboring Balkan populations indicates that Albanian genetic variation follows the broader Southeastern European pattern of isolation by distance and regional continuity rather than discrete population subdivision. These results contribute to the understanding of Albanian population history while simultaneously strengthening the forensic genetic resources available for the region.

## 5. Conclusions

Our findings demonstrate that the Albanian population is characterized by remarkable genetic homogeneity, with negligible population substructure across the northern, central, and southern regions of the country. Comparative analyses further place the Albanian population within the Southeastern European genetic landscape, revealing the strongest affinities with neighboring populations, particularly Greece, Montenegro, and Italy, while maintaining the broader patterns of genetic variation observed across Europe. These results are consistent with the demographic history of the Balkan Peninsula, where repeated migrations, population interactions, and geographic proximity have contributed to a shared genetic background. Beyond providing new insights into the population history of Albania, this study establishes one of the most comprehensive autosomal STR reference datasets currently available for the country. The findings have important implications for forensic genetics, population genetics, and human evolutionary studies by providing a robust reference framework for future comparative analyses and genomic investigations in the Western Balkans. The integration of high-resolution genomic markers and ancient DNA data in future studies will further refine our understanding of the evolutionary processes underlying the genetic landscape of Albania and Southeastern Europe.

## Figures and Tables

**Table 1:** List of populations used for comparative analysis.

Country (Population)	References
Belgium	Decorte et al. (2004) [34]
The Netherlands	Westen et al. (2014) [35]
Norway	Dupuy et al. (2013) [36]
Sweden	Montelius et al. (2008) [37]
Denmark	Pereira et al. (2015) [38]
Ireland and England	Pery et al. (2023) [39]
Finland	Hannelius et al. (2008) [40]
Switzerland	Gehrig et al. (2014) [41]
France	Delest et al. (2020) [42]
Spain	Paredes et al. (2003) [43]
Italy	Piglionica et al. (2013) [44]
Kosovo	Kubat et al. (2004) [16]
Montenegro	Jeran et al. (2007) [13]
Greece	Kovatsi et al. (2006); Sánchez-Diz et al. (2008) [45,46]
Germany	Becker et al. (2006), Seider et al. (2010) [47,48]
Austria	Ross et al. (2001) [49]
Poland	Piatek et al. (2008) [50]
Slovakia	Soták et al. (2008), Červenák et al. (2018) [51,52]
The Czech Republic	Simkova et al. (2009) [53]
Slovenia	Drobnič (2001) [54]
Hungary	Rak et al. (2011) [55]
Croatia	Haliti et al. (2009); Novokmet et al. (2022) [11,18]
Bosnia and Herzegovina	Pilav et al. (2017) [56]
Turkiye	Gurkan et al. (2015); Bozman et al. (2018) [10,57]

## Data Availability

The data supporting the findings of this study are available within the article and its Supplementary Materials. The complete R code and analysis workflow for reproducing all results are publicly available at: https://github.com/ilirsheraj/Albania-STRAF (accessed on 15 July 2026).

## Supplementary Materials

The following supporting information can be downloaded at: https://www.mdpi.com/article/10.3390/genes17091059/s1, Table S1: Distribution of STR allele frequencies across 4000 alleles obtained from 2000 Albanian individuals; Table S2: Summary statistics: Table S3: LD matrix center Albania; Table S4: LD matrix South Albania; Table S5: LD matrix North Albanitaa; Table S6: Nei distance matrix between populations; Table S7: HWE per locus 10k permutations.

## References

[1] Cavalli-Sforza L.L., Menozzi P., Piazza A. (1994). The History and Geography of Human Genes.

[2] Nei M., Kumar S. (2000). Molecular Evolution and Phylogenetics.

[3] Vickers M. (2001). The Albanians: A Modern History.

[4] Wilkes J. (1992). The Illyrians. The Peoples of Europe.

[5] Davranoglou L.-R., Lauka A., Aristodemou A., Maróti Z., Bojaxhi G., Muhaj A., Mikerezi I., Wesolowski D., Joseph B.D., Heraclides A. (2026). Ancient DNA Evidence for the History of the Albanians. Nat. Hum. Behav..

[6] Davranoglou L.-R., Lauka A., Aristodemou A., Maróti Z., Bojaxhi G., Muhaj A., Mikerezi I., Wesolowski D., Joseph B.D., Heraclides A. (2023). Ancient DNA Reveals the Origins of the Albanians. bioRxiv.

[7] Sulcebe G., Sanchez-Mazas A., Tiercy J.-M., Shyti E., Mone I., Ylli Z., Kardhashi V. (2009). HLA Allele and Haplotype Frequencies in the Albanian Population and Their Relationship with the Other European Populations. Int. J. Immunogenet..

[8] Xhetani M., Seferi I. (2014). Distribution of Rhesus Blood Group Antigens and Weak D Alleles in the Population of Albania. Blood Transfus..

[9] Dogan S., Kovacević L., Marjanović D. (2013). Genetic Polymorphisms of 15 STR Loci within Turkish Student Population Living in Sarajevo, Bosnia and Herzegovina. Coll. Antropol..

[10] Gurkan C., Demirdov D.K., Yamaci R.F., Sevay H. (2015). Population Genetic Data for 15 Autosomal STR Markers in Turkish Cypriots from Cyprus. Forensic Sci. Int. Genet..

[11] Haliti N., Carapina M., Masić M., Strinović D., Klarić I.M., Kubat M. (2009). Evaluation of Population Variation at 17 Autosomal STR and 16 Y-STR Haplotype Loci in Croatians. Forensic Sci. Int. Genet..

[12] Havaš D., Jeran N., Efremovska L., Đorđević D., Rudan P. (2007). Population Genetics of 15 AmpflSTR Identifiler Loci in Macedonians and Macedonian Romani (Gypsy). Forensic Sci. Int..

[13] Jeran N., Havas D., Ivanović V., Rudan P. (2007). Genetic Diversity of 15 STR Loci in a Population of Montenegro. Coll. Antropol..

[14] Klaric I.M. (2000). Population Structure of the Rural Communities on the Island of Krk (Croatia): A Comparison of Genetic, Cultural, and Geographic Data. Am. J. Hum. Biol..

[15] Klaric I.M., Pericic M., Lauc L.B., Janicijevic B., Kubat M., Pavicic D., Rudan I., Wang N., Jin L., Chakraborty R. (2005). Genetic Variation at Nine Short Tandem Repeat Loci Among Islanders of the Eastern Adriatic Coast of Croatia. Hum. Biol..

[16] Kubat M., Škavić J., Behluli I., Nuraj B., Bekteshi T., Behluli M., Martinović Klarić I., Peričić M. (2004). Population Genetics of the 15 AmpFlSTR Identifiler Loci in Kosovo Albanians. Int. J. Leg. Med..

[17] Novković T., Panić B., Banjac A., Đekić T.K., Tomišić-Kosić I., Vučetić-Dragović A., Stamenković G., Blagojević J., Marjanović D., Pojskić N. (2010). Genetic Polymorphisms of 15 AmpFlSTR Identifiler Loci in a Serbian Population. Forensic Sci. Int. Genet..

[18] Novokmet N., Marjanović D., Škaro V., Projić P., Lauc G., Grahovac B., Ostojić S., Kapović M., Rudan P. (2011). Genetic Polymorphisms of 15 STR Loci in the Population of the Island of Cres (Croatia). Ann. Hum. Biol..

[19] Novokmet N., Galov A., Škaro V., Projić P., Šarac J., Havaš Auguštin D., Rudan P., Primorac D., Marjanović D. (2022). Genetic Sub-Structuring of Croatian Island Populations in the Southeastern European Context: A Meta-Analysis. Croat. Med. J..

[20] Takić Miladinov D., Vasiljević P., Šorgić D., Podovšovnik Axelsson E., Stefanović A. (2020). Allele Frequencies and Forensic Parameters of 22 Autosomal STR Loci in a Population of 983 Individuals from Serbia and Comparison with 24 Other Populations. Ann. Hum. Biol..

[21] Klarić I.M., Barać L., Buković D., Furač I., Geber G., Janićijević B., Kubat M., Peričić M., Pupić B.V., Rudan P. (2001). Short Tandem Repeat (STR) Variation in Eight Village Populations of the Island of Korčula (Croatia). Ann. Hum. Biol..

[22] Zaimi E., Xhetani M., Haxhia A. (2025). GENETIC VARIATION FOR 16 AUTOSOMAL STR LOCI IN A POPULATION SAMPLE FROM ALBANIA. Int. J. Ecosyst. Ecol. Sci..

[23] (2009). Council Resolution of 30 November 2009 on the Exchange of DNA Analysis Results.

[24] Lerch M., Beckendorff D., Du W. (2025). The Diffusion of International Migration in Cities and Rural Areas of Developing Countries. Int. Migr. Rev..

[25] (2021). Regional Statistical Yearbook, 2021.

[26] Kamvar Z.N., Brooks J.C., Grünwald N.J. (2015). Novel R Tools for Analysis of Genome-Wide Population Genetic Data with Emphasis on Clonality. Front. Genet..

[27] Bougeard S., Dray S. (2018). Supervised Multiblock Analysis in R with the Ade4 Package. J. Stat. Soft..

[28] Weir B.S., Cockerham C.C. (1984). ESTIMATING F-STATISTICS FOR THE ANALYSIS OF POPULATION STRUCTURE. Evolution.

[29] Excoffier L., Smouse P.E., Quattro J.M. (1992). Analysis of Molecular Variance Inferred from Metric Distances among DNA Haplotypes: Application to Human Mitochondrial DNA Restriction Data. Genetics.

[30] Pritchard J.K., Stephens M., Donnelly P. (2000). Inference of Population Structure Using Multilocus Genotype Data. Genetics.

[31] Evanno G., Regnaut S., Goudet J. (2005). Detecting the Number of Clusters of Individuals Using the Software STRUCTURE: A Simulation Study. Mol. Ecol..

[32] Gouy A., Zieger M. (2017). STRAF-A Convenient Online Tool for STR Data Evaluation in Forensic Genetics. Forensic Sci. Int. Genet..

[33] Paradis E., Schliep K. (2019). Ape 5.0: An Environment for Modern Phylogenetics and Evolutionary Analyses in R. Bioinformatics.

[34] Decorte R., Engelen M., Larno L., Nelissen K., Gilissen A., Cassiman J.-J. (2004). Belgian Population Data for 15 STR Loci (AmpFlSTR SGM Plus and AmpFlSTR Profiler PCR Amplification Kit). Forensic Sci. Int..

[35] Westen A.A., Kraaijenbrink T., Robles De Medina E.A., Harteveld J., Willemse P., Zuniga S.B., Van Der Gaag K.J., Weiler N.E.C., Warnaar J., Kayser M. (2014). Comparing Six Commercial Autosomal STR Kits in a Large Dutch Population Sample. Forensic Sci. Int. Genet..

[36] Dupuy B.M., Stenersen M., Kling D. (2013). Frequency Data for 35 Autosomal STR Markers in a Norwegian, an East African, an East Asian and Middle Asian Population and Simulation of Adequate Database Size. Forensic Sci. Int. Genet. Suppl. Ser..

[37] Montelius K., Karlsson A.O., Holmlund G. (2008). STR Data for the AmpFlSTR Identifiler Loci from Swedish Population in Comparison to European, as Well as with Non-European Population. Forensic Sci. Int. Genet..

[38] Pereira V., Tomas C., Sanchez J.J., Syndercombe-Court D., Amorim A., Gusmão L., Prata M.J., Morling N. (2015). The Peopling of Greenland: Further Insights from the Analysis of Genetic Diversity Using Autosomal and X-Chromosomal Markers. Eur. J. Hum. Genet..

[39] Perry J., Matute S.P., Cummings S., Munshi T., Iyavoo S. (2023). English and Irish Population Comparison Using STR Markers: Insights into Genetic Disparities and Historical Influences. Forensic Sci. Int. Rep..

[40] Hannelius U., Salmela E., Lappalainen T., Guillot G., Lindgren C.M., von Döbeln U., Lahermo P., Kere J. (2008). Population Substructure in Finland and Sweden Revealed by the Use of Spatial Coordinates and a Small Number of Unlinked Autosomal SNPs. BMC Genet..

[41] Gehrig C., Balitzki B., Kratzer A., Cossu C., Malik N., Castella V. (2014). Allelic Proportions of 16 STR Loci-Including the New European Standard Set (ESS) Loci-in a Swiss Population Sample. Int. J. Leg. Med..

[42] Delest A., Godfrin D., Chantrel Y., Ulus A., Vannier J., Faivre M., Hollard C., Laurent F.-X. (2020). Sequenced-Based French Population Data from 169 Unrelated Individuals with Verogen’s ForenSeq DNA Signature Prep Kit. Forensic Sci. Int. Genet..

[43] Paredes M., Crespillo M., Luque J.A., Valverde J.L. (2003). STR Frequencies for the PowerPlex 16 System Kit in a Population from Northeast Spain. Forensic Sci. Int..

[44] Piglionica M., Lonero Baldassarra S., Giardina E., Tonino Marsella L., Resta N., Dell’Erba A. (2013). Allele Frequencies of the New European Standard Set (ESS) Loci in a Population of Apulia (Southern Italy). Forensic Sci. Int. Genet..

[45] Sánchez-Diz P., Menounos P.G., Carracedo A., Skitsa I. (2008). 16 STR Data of a Greek Population. Forensic Sci. Int. Genet..

[46] Kovatsi L., Parsons T.J., Just R.S., Irwin J.A. (2006). Genetic Variation for 15 Autosomal STR Loci (PowerPlex 16) in a Population Sample from Northern Greece. Forensic Sci. Int..

[47] Seider T., Fimmers R., Betz P., Lederer T. (2010). Allele Frequencies of the Five miniSTR Loci D1S1656, D2S441, D10S1248, D12S391 and D22S1045 in a German Population Sample. Forensic Sci. Int. Genet..

[48] Becker D., Vogelsang D., Brabetz W. (2006). Evaluation of Seven Autosomal STR Loci in a German Population. Int. Congr. Ser..

[49] Ross J., Parson W., Furać I., Kubat M., Holland M. (2001). Multiplex PCR Amplification of Eight STR Loci in Austrian and Croatian Caucasian Populations. Int. J. Leg. Med..

[50] Piatek J., Jacewicz R., Ossowski A., Parafiniuk M., Berent J. (2008). Population Genetics of 15 Autosomal STR Loci in the Population of Pomorze Zachodnie (NW Poland). Forensic Sci. Int. Genet..

[51] Soták M., Petrejčíková E., Bernasovská J., Bernasovský I., Sovičová A., Boroňová I., Švičková P., Bôžiková A., Gabriková D. (2008). Genetic Variation Analysis of 15 Autosomal STR Loci in Eastern Slovak Caucasian and Romany (Gypsy) Population. Forensic Sci. Int. Genet..

[52] Červenák Z., Mikula M., Matúšek J., Ferák V., Choma A. (2018). Population Genetic Data for 16 STR Loci in Slovakia. Leg. Med..

[53] Simková H., Faltus V., Marvan R., Pexa T., Stenzl V., Broucek J., Horínek A., Mazura I., Zvárová J. (2009). Allele Frequency Data for 17 Short Tandem Repeats in a Czech Population Sample. Forensic Sci. Int. Genet..

[54] Drobnič K., Regent A., Budowle B. (2001). STR Data for the AmpFlSTR SGM plus from Slovenia. Forensic Sci. Int..

[55] Rak S.Á., Zalán A., Szabados G., Pamjav H. (2011). Population Genetic Data on 15 STR Loci in the Hungarian Population. Forensic Sci. Int. Genet..

[56] Pilav A., Pojskić N., Ahatović A., Džehverović M., Čakar J., Marjanović D. (2017). Allele Frequencies of 15 STR Loci in Bosnian and Herzegovinian Population. Croat. Med. J..

[57] Bozman N., Gurkan C., Sevay H., Demirdov D.K., Ozbas-Gerceker F. (2018). Genetic Characterisation of 19 Autosomal STR Loci in a Population Sample from the Southeastern Anatolia Region of Turkey. Ann. Hum. Biol..

[58] Peričić M., Lauc L.B., Klarić I.M., Rootsi S., Janićijević B., Rudan I., Terzić R., Čolak I., Kvesić A., Popović D. (2005). High-Resolution Phylogenetic Analysis of Southeastern Europe Traces Major Episodes of Paternal Gene Flow Among Slavic Populations. Mol. Biol. Evol..

[59] Battaglia V., Fornarino S., Al-Zahery N., Olivieri A., Pala M., Myres N.M., King R.J., Rootsi S., Marjanovic D., Primorac D. (2009). Y-Chromosomal Evidence of the Cultural Diffusion of Agriculture in Southeast Europe. Eur. J. Hum. Genet..

[60] Kovacevic L., Tambets K., Ilumäe A.-M., Kushniarevich A., Yunusbayev B., Solnik A., Bego T., Primorac D., Skaro V., Leskovac A. (2014). Standing at the Gateway to Europe-The Genetic Structure of Western Balkan Populations Based on Autosomal and Haploid Markers. PLoS ONE.

[61] Marjanovic D., Kapur L., Drobnic K., Budowle B., Pojskic N., Hadziselimovic R. (2004). Comparative Study of Genetic Variation at 15 STR Loci in Three Isolated Populations of the Bosnian Mountain Area. Hum. Biol..

[62] Capelli C., Redhead N., Romano V., Calì F., Lefranc G., Delague V., Megarbane A., Felice A.E., Pascali V.L., Neophytou P.I. (2006). Population Structure in the Mediterranean Basin: A Y Chromosome Perspective. Ann. Hum. Genet..

[63] Fiorito G., Di Gaetano C., Guarrera S., Rosa F., Feldman M.W., Piazza A., Matullo G. (2016). The Italian Genome Reflects the History of Europe and the Mediterranean Basin. Eur. J. Hum. Genet..

[64] Koptekin D., Aydoğan A., Karamurat C., Altınışık N.E., Vural K.B., Kazancı D.D., Doğu A.K., Kaptan D., Gemici H.C., Yüncü E. (2025). Out-of-Anatolia: Cultural and Genetic Interactions during the Neolithic Expansion in the Aegean. Science.

[65] Salmela E., Lappalainen T., Fransson I., Andersen P.M., Dahlman-Wright K., Fiebig A., Sistonen P., Savontaus M.-L., Schreiber S., Kere J. (2008). Genome-Wide Analysis of Single Nucleotide Polymorphisms Uncovers Population Structure in Northern Europe. PLoS ONE.

[66] Palo J.U., Ulmanen I., Lukka M., Ellonen P., Sajantila A. (2009). Genetic Markers and Population History: Finland Revisited. Eur. J. Hum. Genet..

[67] Li Z., Gastwirth J.L. (2004). On the Power of Affected Relative Pair Designs for Linkage Studies. Ann. Hum. Genet..

[68] Butler J.M. (2015). Advanced Topics in Forensic DNA Typing: Interpretation.

